# Supplementary figures and images for: In Search for Low-Molecular-Weight Ligands of Human Serum Albumin That Affect Its Affinity for Monomeric Amyloid β Peptide
Source: Int J Mol Sci. 2024 May 2;25(9):4975. doi: 10.3390/ijms25094975 (PMC11084196; doi:10.3390/ijms25094975)

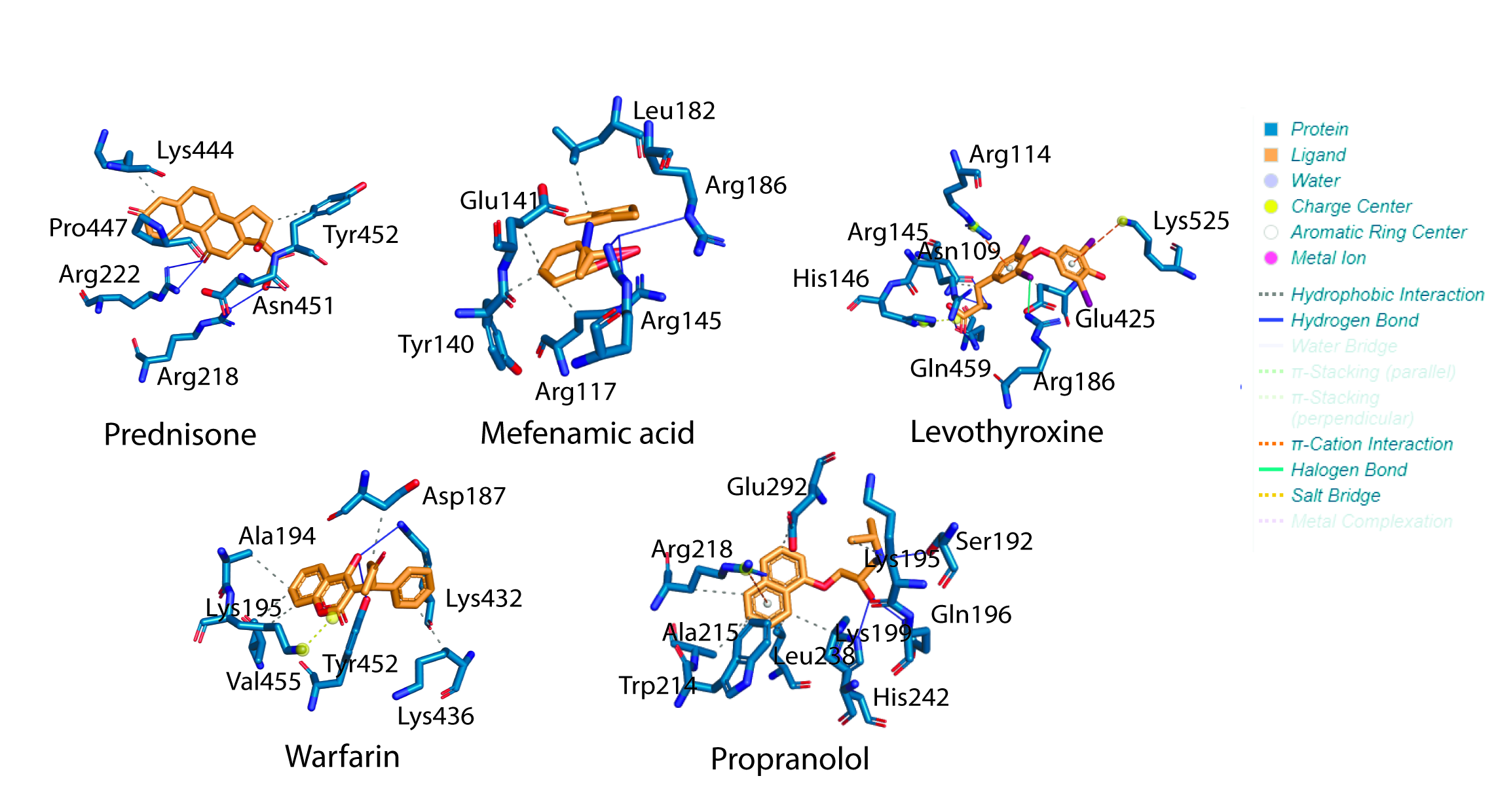

Supplement: Supplementary file 1 [file ijms-25-04975-s001.zip › Figure S1.tif]
